# Solid Oxide Electrochemical Systems: Material Degradation Processes and Novel Mitigation Approaches

**DOI:** 10.3390/ma11112169

**Published:** 2018-11-02

**Authors:** Michael Reisert, Ashish Aphale, Prabhakar Singh

**Affiliations:** Department of Materials Science and Engineering, University of Connecticut, Storrs, CT 06269, USA; michael.reisert@uconn.edu (M.R.); ashish.aphale@uconn.edu (A.A.)

**Keywords:** corrosion, electrode poisoning, solid oxide, interconnect, electrode, oxide scale

## Abstract

Solid oxide electrochemical systems, such as solid oxide fuel cells (SOFC), solid oxide electrolysis cells (SOEC), and oxygen transport membranes (OTM) enable clean and reliable production of energy or fuel for a range of applications, including, but not limited to, residential, commercial, industrial, and grid-support. These systems utilize solid-state ceramic oxides which offer enhanced stability, fuel flexibility, and high energy conversion efficiency throughout operation. However, the nature of system conditions, such as high temperatures, complex redox atmosphere, and presence of volatile reactive species become taxing on solid oxide materials and limit their viability during long-term operation. Ongoing research efforts to identify the material corrosion and degradation phenomena, as well as discover possible mitigation techniques to extend material efficiency and longevity, is the current focus of the research and industrial community. In this review, degradation processes in select solid oxide electrochemical systems, system components, and comprising materials will be discussed. Overall degradation phenomena are presented and certain degradation mechanisms are discussed. State-of-the-art technologies to mitigate or minimize the above-mentioned degradation processes are presented.

## 1. Introduction

### 1.1. Solid Oxide Electrochemical Systems

There is an ever-growing push to utilize alternative, clean forms of energy and deviate from a dependency on nonrenewable fossil fuels. One way this has been achieved is through the exploitation of electrochemical devices, which convert the chemical energy from fuels into electricity or vis-versa without any combustion of the fuels [1]. Some systems are currently integrated in electrical grids and used in automotive/aerospace applications as liquid or polymer electrolytes. However, a different variety is comprised of a solid metal oxide electrolyte and electrodes. These solid oxide electrochemical systems are favorable as they provide high energy conversion efficiencies, flexibility in design, and flexibility in fuel choice [2,3,4]. 

This review will discuss three particular solid oxide electrochemical systems: Solid oxide fuel cells, solid oxide electrolysis cells, and oxygen transport membranes, as well as related degradation processes associated with them. To achieve wide-scale industrial use of solid oxide electrochemical devices, the materials that comprise them and any material degradation processes must be well understood. Major degradation phenomena can occur at every system component. In this review, the solid–gas interactions which play a large role in system performance degradation will be discussed [5,6]. This includes poisoning of both fuel and air electrodes by volatilized species, as well as oxidation and metal loss, resulting from simultaneous exposure of metallic components to different gaseous species. Knowledge of these phenomena and a review of the current state-of-the-art technology and novel mitigation approaches will be useful in prolonging material lifespans for efficient operation of solid oxide electrochemical systems.

#### 1.1.1. Solid Oxide Fuel Cells

Fuel cells are open systems that can be continuously fueled. This makes fuel cells optimal for grid applications, as they can be intermittently refueled to continuously provide electricity with no system replacement necessary [7,8]. They operate more efficiently than thermomechanical means of energy production as direct energy conversion eliminates the need for combustion [4]. Fuel cells have been designed with various materials, specifically electrolyte materials, to yield a variety of types. A proton exchange membrane fuel cell (PEMFC), for example, utilizes a polymer electrolyte such as Nafion^®^ to foster protonic movement. A platinum catalyst is used as an anode to split the hydrogen fuel into protons and electrons. The electrons are forced to an external circuit due to the electrically insulating properties of the electrolyte, while the protons are conducted through the electrolyte toward the cathode. At the cathode, the protons are reunited with electrons that have travelled the external circuit, as well as oxygen from an oxidizing gas flown to the cathode. This results in the chemical formation of water vapor, which is filtered out of the cell as waste. This electrochemistry is the driving force behind fuel cell operation and has been adhered to in developing new types of fuel cells.

**Figure 1:**
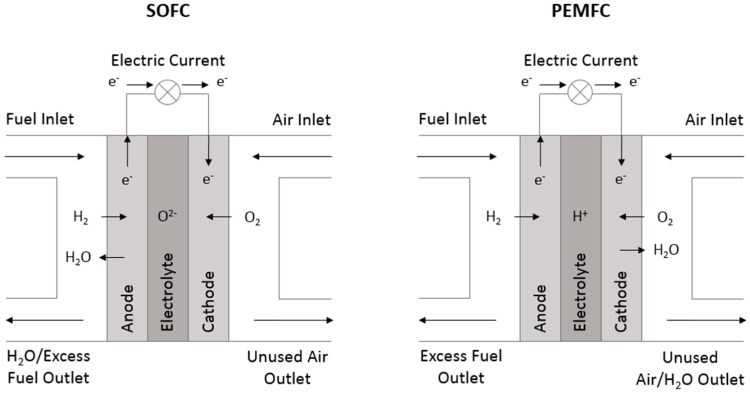
Comparative operative schematic of a solid oxide fuel cell (SOFC) and proton exchange membrane fuel cell (PEMFC).

One promising variety of fuel cell is the solid oxide fuel cell (SOFC), which uses a solid ceramic electrolyte. This is advantageous as the ceramic electrolyte is very stable and offers a long operating lifetime, whereas polymer electrolytes can dry out or flood if they are not hydrated in the precise amount, at which point they lose efficiency or stop working altogether [9]. They can also operate on a range of hydrogen-based fuels like hydrocarbons, whereas PEM fuel cells must use a pure hydrogen fuel source [4]. Solid oxide fuel cells operate at high temperatures by reducing an oxidizing gas (usually air) at the cathode into oxygen ions. Unlike PEM fuel cells, which reduce a fuel and move ions from anode to cathode, a SOFC moves oxygen ions from cathode to anode, where they meet hydrogen ions to form water vapor as waste and release an electron to an external circuit. This is because of the nature of the solid electrolyte, which is typically an oxygen-ion conducting yttria-stabilized zirconia (YSZ) ceramic [2,3]. The cathode, also known as the air electrode (AE), utilizes electronically conducting or mixed ionically and electronically conducting, oxygen-reducing ceramics, which have a thermal expansion coefficient that closely matches the electrolyte to reduce thermal stresses under dynamic operating conditions. A comparative schematic of the operating principles of a SOFC and a PEMFC is pictured below in Figure 1.

While typically operating on the principle of oxygen ion conduction, some SOFC systems can produce energy through proton conduction similar to that shown in the PEMFC operation [10,11,12,13]. The materials which enable this are discussed later in this review.

#### 1.1.2. Solid Oxide Electrolysis Cells

**Figure 2:**
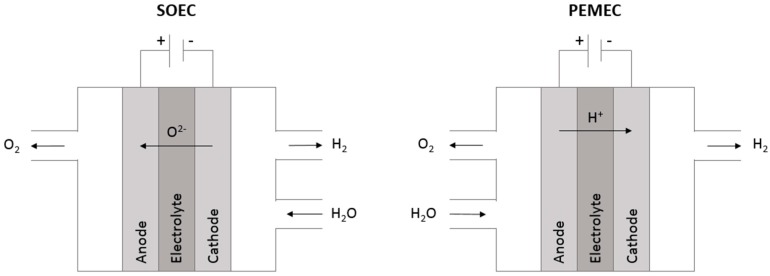
Comparative schematics of a polymer exchange membrane electrolysis cell (PEMEC) and a solid oxide electrolysis cell (SOEC) (Based on schematics from [15]).

The governing principles of electrochemistry that allow fuel cells to efficiently produce electricity can also be used to produce fuel or oxygen by means of electrolysis. Electrolysis is the splitting of gaseous species, such as water vapor or carbon dioxide, into their constituents through the application of an external voltage. Essentially, a fuel cell can be operated in reverse to create what is called an electrolysis cell [14]. The reactions that occur within an electrolysis cell are non-spontaneous redox reactions and require electrical energy in the form of an applied voltage to proceed. Therefore, the function of these systems is to convert electrical energy to chemical energy. In the case of water electrolysis, which is carried out to create hydrogen for fuel, both polymer exchange membrane electrolysis cells (PEMEC) and solid oxide electrolysis cells (SOEC) can be used [15]. In a PEMEC, water is split at the anode in what is called an oxygen evolution reaction (OER). It is oxidized into oxygen gas, protons, and electrons. The cathode undergoes a hydrogen evolution reaction (HER), where the protons from the anode travel through the electrolyte and meet the supplied electrons to create hydrogen gas [15]. For the SOEC, the electrolyte is oxygen conducting, as it is in the solid oxide fuel cell. Therefore, water is split at the cathode and oxygen ions travel to the anode where they release electrons [14,15]. The supply of electrons to the cathode deprotonates the hydrogen and creates hydrogen gas. The co-electrolysis of CO_2_ and H_2_O using SOEC technology is a promising means of hydrocarbon fuel production, while recycling greenhouse gas emissions [14,16]. A comparative schematic of the operating principles of a SOEC and PEMEC is pictured below in Figure 2.

#### 1.1.3. Oxygen Transport Membranes

**Figure 3:**
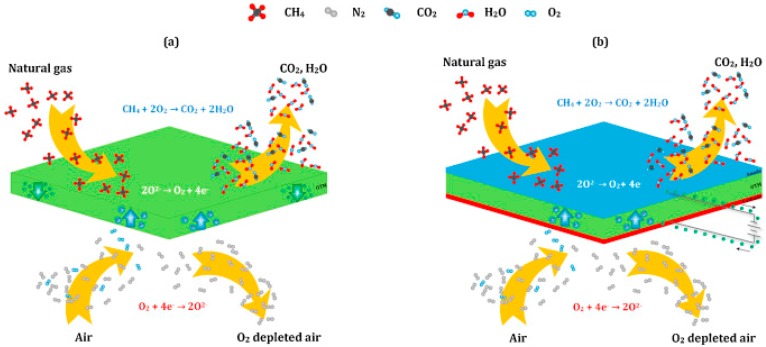
Comparative schematic of: (**a**) a mixed ionic-electronic conductor (MIEC) and (**b**) an ionic conductor, both used as oxygen transport membranes, adapted from [17], with permission from Elsevier, 2018.

Oxygen transport membranes (OTM) are used in separating high-purity oxygen from air. This is used in the oxy-combustion process, where high purity oxygen is used to ignite fuel. The oxy-combustion process is more efficient and cleaner than the standard means of using air as an oxidant. Solid oxide membranes can be either ionic conductors or mixed ionic electronic conductors (MIEC), as shown in Figure 3. Ionic conductors are called passive membranes as they are electrically driven by an outside source [17]. The MIEC membrane is called an active membrane and it relies on the difference of oxygen partial pressure (*P*O_2_) on either side of the membrane [17]. Oxygen-rich air yields a high *P*O_2_ on one side of the membrane. A natural gas flown on the opposing side results in a much lower *P*O_2_, and thermodynamically, oxygen ions are driven toward the lower *P*O_2_.

### 1.2. Device and System Materials

Solid oxide electrochemical systems are most often comprised of three major components; an anode, a cathode, and an electrolyte. In the case of OTM’s, configurations with these three components or one single membrane acting as an electrolyte are possible. System components work harmoniously during chemical reactions to provide the desired end goal of either electrical energy or pure gaseous species. The metals and metal oxides used for these system components must fulfill several criteria for efficient cell operation, such as conductivity of either ions, electrons, or both species, matching coefficients of thermal expansion, and strong catalytic activity.

#### 1.2.1. Electrolytes

The most important electrochemical system component is the electrolyte. In solid oxide fuel and electrolysis cells, ceramic metal oxides are used to conduct either oxygen ions or protons. Yttria-stabilized zirconia (YSZ) is valued as an oxygen ion electrolyte material, and is commonly used in high-temperature applications [2,3,4]. This value is due to the ceramic’s high oxygen ion conductivity, diffusivity, and thermal stability. These properties all arise from the cubic fluorite structure developed when yttria (Y_2_O_3_) is used as a dopant within the zirconia (ZrO_2_) structure. The cubic phase of zirconia is not stable at room temperature because Zr^4+^ ions are too small to stabilize this phase, and instead the monoclinic phase is observed. However, doping the structure with a slightly larger cation, most often yttrium ions (Y^3+^), promotes the formation of the cubic fluorite structure at much lower temperatures down to room temperature. This doping creates oxygen vacancies which allow for transport of oxygen ions, following the Kröger-Vink reaction in Equation (1) [18]:

Y_2_O_3_ → 2Y′_Zr_ + 3O^x^_O_ + V^∙∙^_O_ (1)

**Figure 4:**
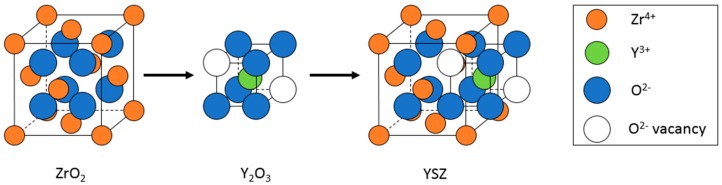
Cubic structure of yttria-stabilized zirconia (YSZ).

A pictorial representation of the defect structure of YSZ is shown below in Figure 4.

Other oxygen-conducting ceramics used as electrolytes include doped cerium oxides like samarium doped ceria (CSO or SDC) and gadolinia-doped ceria (CGO or GDC), which offer high oxygen ion conductivities at intermediate temperatures (500–700 °C) [4]. However, they are not suited for high temperatures as reducing atmosphere at elevated temperature causes a partial reduction of ceria to Ce^3+^, which makes the electrolyte electronically conductive and greatly reduces efficiency [19]. For oxygen transport membranes, mixed electronic-ionic conductors like many lanthanum-based perovskites are used as electrolytes, as they allow pure oxygen ion conduction driven by an oxygen partial pressure (*P*O_2_) difference [17]. These perovskites, often used as electrode materials, will be further described in the following section. The passive or electrically driven OTM uses a pure ionic conductor like YSZ and operates much like a SOEC.

Proton-conducting SOFC/SOEC systems typically use barium-based perovskites of the structure Ba(Ce,Y)O_3-δ_ (BCY) as electrolyte materials [10,12,13]. Typical dopants of this structure include Sr, Zr, Yb, and Fe [13]. These dopants can increase protonic conductivity and stability for implementation within proton-conducting SOFC/SOEC systems [20].

#### 1.2.2. Electrodes

SOFC and SOEC electrodes refer to the anode, or fuel electrode, and cathode, or air electrode. These electrodes are the sites of certain reactions which allow ionic transfer through the electrolyte. Both electrode types must maintain favorable reactivity at the triple-phase boundary (TPB) of the electrode, electrolyte, and gaseous species [21,22]. The cathode must have adequate porosity to allow gaseous oxygen to diffuse toward the electrolyte, as well as high electronic conductivity, to allow reduction of oxygen [21]. Cathode materials are often perovskite-type oxides of the general formula ABO_3_ [21,23]. The A site cation can be a mix of rare and alkaline earth metals, whilst the B site is a transition metal which enables catalysis for the redox (reduction-oxidation) reaction at the cathode [21]. Therefore, doping of these cation sites can enable better electronic conductivity and electrocatalytic properties [21]. Many variations of lanthanum-based perovskites exhibit good cathodic properties. These lanthanum-based oxides are often doped at the A site with Sr, which produces increased electron-hole concentrations and reduces unwanted reactivity of La with electrolyte materials [21]. They are further classified by the B site dopant, which can include Mn, Fe, or Co.

In conventional SOFC systems, the fuel electrode must catalyze the reaction between fuel and oxygen ions from the electrolyte, whilst fostering electronic transfer to the external circuit [24]. Platinum has favorable catalytic properties, which warranted its use and integration into both cathodes and anodes for various solid oxide electrochemical systems [4,13,24]. However, the extremely high cost of platinum has spurred efforts to develop cheaper electrode alternatives [4,24]. Furthermore, nickel metal as an anode material offers lower polarization resistance and a cheaper price compared to platinum [2]. Nickel mixed with the electrolyte material (Ni-YSZ or Ni-BCY) is used to create a porous mixed ionic-electronic conducting cermet that efficiently allows fuel flow toward the electrolyte [2,24]. Since Ni is susceptible to carbide formation, other material choices include composite cermets based on Cu and CGO, which have been considered and show promise in carbonaceous atmospheres [22,24]. Some perovskite-structured ceramics are also used as anode materials because of their mixed conductivities [24].

#### 1.2.3. Interconnects/Sealants/Balance-of-Plant

For proper assembly and gas flow for multiple fuel and electrolysis cell stacks, structural components known as interconnects are relied on. At elevated operating temperatures (800+ °C), LaCrO_3_ ceramics were the primary interconnect material choice [2,3,25,26]. However, advancements in electrolyte materials and dimensions have allowed solid oxide fuel cell operating temperatures to fall to an intermediate range of 600–800 °C [27]. This has opened a window for new materials to be used for interconnects, specifically cheaper and more easily manufactured metal alloys. The ceramic components of a solid oxide fuel cell have coefficients of thermal expansion of around 10^−12^ × 10^−6^ K^−1^ [28]. To ensure the cell does not fail under thermal stress, the interconnect materials contacting the ceramic components must match or nearly-match the thermal expansion of the ceramics. Interconnects are also relied on to assist in the circuit, which ultimately yields the fuel cells electrical voltage output. For this, interconnects must be conductive and resist any ohmic losses during operation. To deviate from the expensive ceramic lanthanum-chromite interconnects that have previously been used, certain iron, chromium, and nickel alloys have been more easily and more cost-effectively manufactured to meet these requirements. The development of these materials specifically for use as interconnects has drawn a lot of attention and inspired many research efforts to maximize their efficiency and compatibility.

Interconnects and other cell components must be hermetically sealed to ensure no fuel or oxidant loss during operation. In planar configurations of SOFC/SOEC/OTM systems, the outer edges of interconnects, electrodes, and electrolytes, as well as between individual cells, are sealed to bond the entire stack [29]. State-of-the-art seal types include rigidly bonded silica glass seals which provide hermetic sealing, flexible design integration, cheap costs, and tailored properties [29].

**Figure 5:**
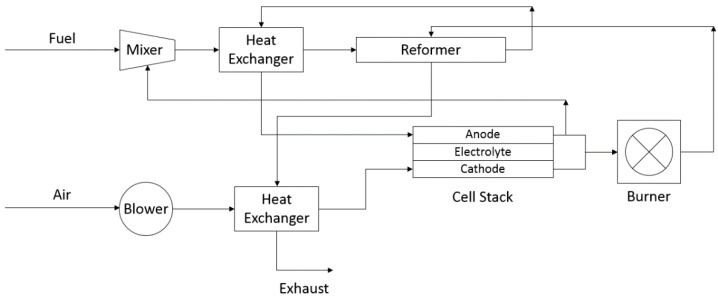
Flow chart of balance-of-plant (BOP) components/system operation (based on schematic from Reference [30]).

Balance-of-plant (BOP) materials include external system components, which mix, heat, reform, and flow gases to the cell stack [30]. These components are needed for all solid oxide electrochemical systems, as they allow properly heated gases to infiltrate respective cell stack components and ensure proper cell operation. A flow chart highlighting the components of BOP with respect to the cell stack is shown below, in Figure 5.

## 2. Material Corrosion/Degradation Phenomena

**Figure 6:**
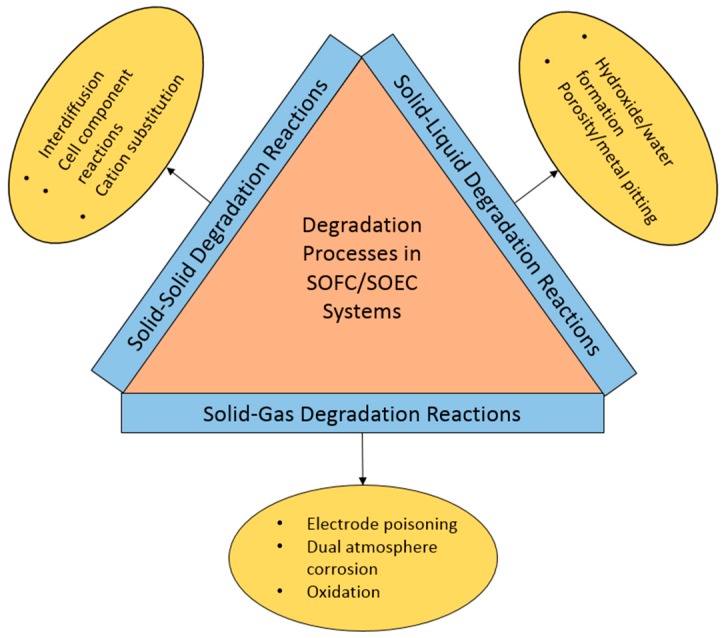
Schematic representation of the overall degradation processes in (solid oxide fuel cells) SOFC/SOEC systems.

Owing to the complexity of solid oxide electrochemical system materials and operating conditions, a variety of degradation processes continue to plague some full-fledged grid integrations and industrial uses. These can occur as solid–solid, solid–gas, or solid–liquid degradation reactions, as shown in Figure 6. As previously mentioned, the interactions between solid materials and the oxidizing or reducing gases in the system are extremely problematic. Two detrimental gas/solid reactions are those that occur at active areas in the cell, and those that lead to the breakdown of structural components. These degradation phenomena will be discussed in detail.

### 2.1. Electrode Poisoning and Degradation

An ongoing issue that has plagued the viability of many solid-state electrochemical devices is the poisoning of electrode species by volatile and atmospheric species. These species are volatized from interconnect and sealant materials at typical operating temperatures and durations of SOFC/SOEC/OTM systems [5,17].

#### 2.1.1. Sulfur

**Figure 7:**
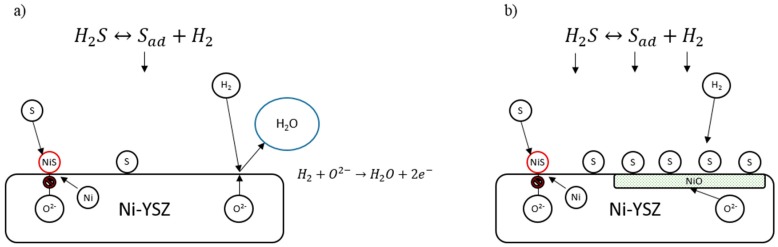
Mechanisms of sulfur poisoning in: (**a**) Low partial pressure of hydrogen sulfide (*P*H_2_S) fuel where sulfur adsorption decreases some active sites by forming NiS; and (**b**) High *P*H_2_S fuel where nickel in the anode oxidizes.

Sulfuric impurities in hydrocarbon fuels such as H_2_S degrade Ni-YSZ and Ni-BZCYYb (Ni-BCY doped with Zr, Yb) electrodes by forming nickel sulfide, which forms as large particles that reduce the triple phase boundary where active ionic species transfer at the electrode/electrolyte interface [31,32]. This degradation may also occur due to sulfur chemisorption on the electrode and saturation of the electrode, which can also reduce active sites [33]. In these poisoning conditions, nickel has also been shown to oxidize, which leads to larger polarization and cell voltage drop, greatly reducing the nickel catalyst’s efficacy (Figure 7) [33].

Sulfur can also exist as an air impurity in the form of SO_2_, and therefore, affect the cathode air stream [34]. At the lanthanum strontium cobalt ferrite (LSCF) air electrode, SO_2_ impurities in the oxidant react with strontium to form SrSO_4_ deposits on the electrode surface, which prevent oxygen diffusion [34]. This has resulted in research and approaches to prevent sulfurous contamination of the cell.

#### 2.1.2. Chromium

Chromium has been shown to volatilize (evaporate) from chromium-containing ferritic steels used for interconnects and in BOP components [35]. Over prolonged operation in a SOFC/SOEC, ferritic steels which form a chromia scale can lose chromium through volatilization (evaporation). Furthermore, chromium species can diffuse through the triple phase boundary and deposit on the air electrode, limiting the oxygen reduction reaction at the air electrode. The chromium species can also react with electrode constituents and create complex oxides, which has been shown to cause cell degradation [36,37,38]. In systems with strontium and manganese, such as LSM cathodes, gaseous chromium species can cause the growth of solid SrCrO_4_ and spinel-type (Cr,Mn)_3_O_4_, which also reduce electrochemical activity by building on active boundary area [35]. Jiang et al. found that deposition of chromium occurred on the LSCF electrode used, while in an interconnect/LSM/YSZ cell, chromium deposited on the electrolyte surface [39]. Each area of deposition was found to reduce cell efficiency by blocking the active area for oxygen reduction at the electrolyte/electrode interphase.

In humid air, CrO_2_(OH)_2_ is the most abundant chromium gaseous species [40]. This gaseous species forms in the following reaction process:

(2) $${\mathrm{Cr}}_{2}O_{3\;\left(s\right)}+\frac{3}{2}O_{2\;\left(g\right)}+2H_{2}O_{\left(g\right)}\to 2{\mathrm{CrO}}_{2}{\left(\mathrm{OH}\right)}_{2\;\left(g\right)}$$

Kurokawa et al. found that the enthalpy of reaction favored this species under the given conditions, which matched previous works and proved that this gaseous chromium species is most abundant and to blame for electrode poisoning [40]. This gaseous species reduces at the triple phase boundary and forms solid Cr_2_O_3_, thereby reducing the active area at the boundary and preventing oxygen reduction [35]. 

#### 2.1.3. Silicon

Silicon impurities are a result of volatilization from glass sealants used to hermetically seal cell stacks. These seals are used in many SOFC/SOEC/OTM systems as separation of gaseous species and leak-proof stacks are critical for efficient system operations. Volatile Si species can deposit at active triple phase boundary sites of the Ni-YSZ electrode/YSZ electrolyte interface as silica (SiO_2_) in SOFC and SOEC systems, and at electrocatalytically active sites in OTM systems [5,17,29,41,42]. Glassy-phase solid SiO_2_ deposits with the equilibration of the following equation, as in References [5,42]:

(3) $$\mathrm{Si}{\left(\mathrm{OH}\right)}_{4}\left(g\right)↔{\mathrm{SiO}}_{2}\left(s\right)+2H_{2}O\;\left(g\right)$$

Gaseous silicon hydroxide (Si(OH)_4_) forms SiO_2_ deposits and steam at the cell areas where steam is reduced to hydrogen. This is most prominent where the hydrogen electrode is closest to the electrolyte, leading to high concentrations of deposited SiO_2_ at the electrode/electrolyte interface which block electrocatalytic sites [5,42]. SiO_2_ deposition at active sites simultaneously leads to increased non-ohmic polarization and ohmic losses at the fuel electrode [29].

### 2.2. Corrosion of Metallic Components

**Figure 8:**
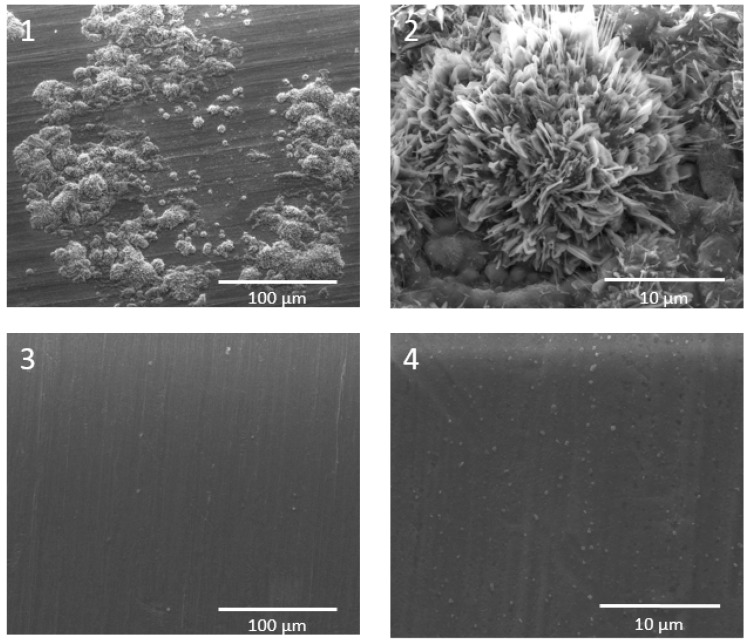
Comparison of ferritic stainless-steel samples in dual atmosphere (**1** and **2**) and single/dry air atmosphere (**3** and **4**).

Interconnect materials in SOFC/SOEC systems are exposed to a so-called dual atmosphere of air and hydrogen-based fuel on opposing sides. They must separate these gases and ensure the gases flow to their respective electrodes for proper cell function. Therefore, interconnects must remain stable in this dual atmosphere for the entire cell lifespan to maintain cell efficiency. However, at the intermediate cell operating temperature, interconnects undergo aggressive corrosion via iron oxidation [43,44]. More specifically, it has been shown experimentally that this oxidation occurs rapidly (within a 50 h test) and is more pronounced on the air-exposed side of the interconnect alloy (Figure 8(1,2)). In comparison, an interconnect alloy left to oxidize in a single atmosphere of air (Figure 8(3,4)) or hydrogen-based fuel for the same operating conditions does not show nearly the expanse and severity of oxidation, highlighting the phenomenon is a result of the specific dual atmosphere condition. The phenomenon occurs as a result of hydrogen permeation in the metal and its movement through the metal and oxide scales, where it contributes to nodule iron oxide growth, and ultimately platelet-like outward growth. It also appears to occur more severely at lower operating temperatures. Alnegren et al. tested AISI 441 in a dual atmosphere exposure at 600, 700, and 800 °C, and found that more severe dual atmosphere corrosion occurred at 600 °C. The samples at higher temperatures formed the more preferred chromia/MnCr_2_O_4_ spinel scales with little iron oxide overgrowth. This “inverse temperature” effect is not well known [44].

## 3. Mitigation Approaches

To suppress material degradation, certain state-of-the-art methods have been developed. These methods, along with some newer means of prolonging material lifetimes will be discussed. Current state-of-the-art mitigation methods include coating metallic components to reduce species volatilization, and doping electrode materials to prevent poisonous species deposition. Other more novel approaches include the use of getters to remove certain volatile species. These forms of degradation mitigation will now be further discussed with respect to certain component degradations aforementioned.

### 3.1. Electrode Poisoning Mitigation

**Figure 9:**
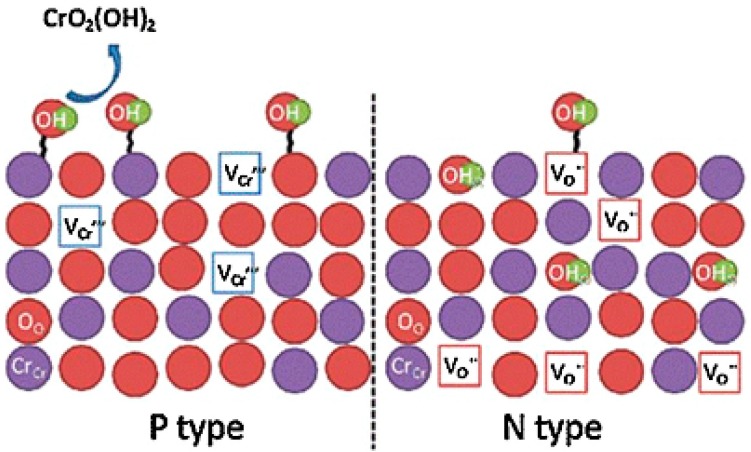
Comparison of defect structures of p-type and n-type chromia with regards to surface adherence of OH^−^ groups, adapted from [45], with permission from Elsevier, 2018.

In pre-oxidizing AISI 441 stainless steel in a CO_2_/CO mix at 850 °C, Wongpromrat et al. found that the pre-oxidized alloy did not evolve as much gaseous chromium species due to the promotion of a single n-type chromia scale influenced by the low oxygen partial pressure pre-oxidizing atmosphere of 10^−10^ bar *P*O_2_ [45]. The defect scale of this chromia type lowered adherence of water vapor chromium species and ultimately lowered the volatilization rate [45]. This is because the possible point defects within the n-type structure are oxygen vacancies and chromium interstitials [45,46]. Therefore, the OH^-^ groups can become dissolved in the oxide which is preferred, as the OH^-^ groups adsorbed on the outside are easily combined with chromia to form the gaseous species CrO_2_(OH)_2_. Chromia scales grown in air or oxygen, with a higher oxygen partial pressure, were a mix of p-type and n-type in defect structure, and therefore, exhibited more adsorbed OH^-^ groups as depicted in Figure 9 [45,46].

**Figure 10:**
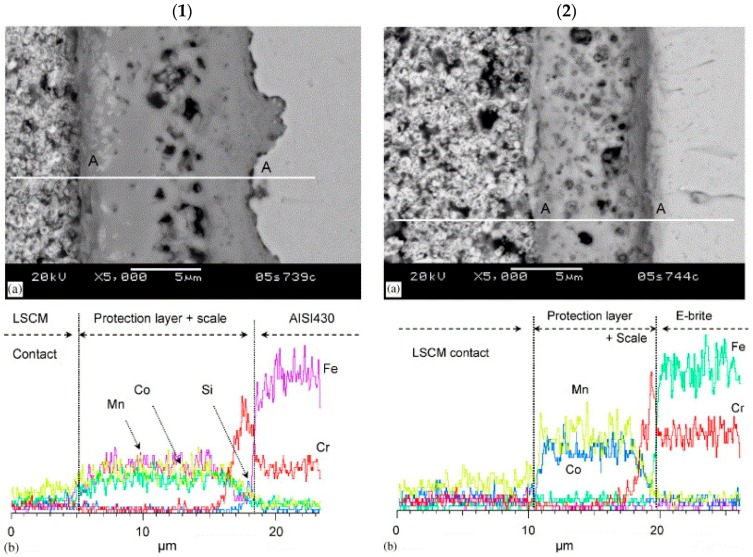
SEM/EDS analysis of: (**1**) AISI430 and (**2**) E-brite with Mn_1.5_Co_1.5_O_4_ protection layer after the contact ASR measurement at 800 °C in air for about 400 h. (**a**) SEM cross-section and (**b**) EDS line scan along line A–A in (**a**), adapted from [49], with permission from Elsevier, 2018.

Reactive element oxides are not sufficient in preventing evaporation as they are typically porous and thin in nature [47]. Likewise, perovskite coatings have proven ineffective as a chromium barrier [48]. The spinel-type oxides have proven to be a much more effective coating variety in suppressing chromium evaporation. Yang et al. used a slurry-coating technique to deposit a spinel Mn_1.5_Co_1.5_O_4_ coating on the ferritic stainless steels AISI 430 and E-brite [49]. In Figure 10, both AISI 430 and E-brite were coated and the area-specific resistance (ASR) was measured at 800 °C for 400 h.

At the conclusion of the test, SEM and EDS were used to characterize the interconnect materials. It was found that no chromium species reached the LSCM cathode, revealing the efficacy of the coating [49].

In an attempt to mitigate the effects of sulfur poisoning on cell anodes, Marina et al. added antimony and tin to Ni-YSZ electrodes and showed a reduction in sulfur adsorption. This can been accredited to Sb and Sn taking up active sites at the electrode surface, weakened sulfur adsorptive bonds, increased sulfur oxidation, or secondary phase formation [50]. What seems most likely is that, since Sb and Sn segregate to surface grain boundaries (the more active surface sites), it may directly prohibit adsorption of sulfur at those sites, much like sulfur prohibits hydrogen from adhering during poisoning [50]. More work is needed to determine the exact mechanism of these added metallic impurities. However, despite an initial decrease in hydrogen oxidation of the Sb and Sn-doped electrodes, which recovered after these impurities diffused into the bulk Ni-YSZ, the inclusion of Sb and Sn in the electrode has a positive mitigating effect against sulfur poisoning.

Recently, mitigation approaches to chromium poisoning of cell cathodes have led to the development of chromium getters. Aphale et al. tested the stability of a SrO-NiO, or Sr_x_Ni_y_O, solid solution getter, which was shown to remain stable up to 900 °C [51]. This getter composition had been shown to be effective in capturing gaseous chromium species through the following reaction, as in Reference [52]:

(4) $${\mathrm{Sr}}_{9}{\mathrm{Ni}}_{7}O_{21\left(s\right)}+{\mathrm{CrO}}_{2}{\left(\mathrm{OH}\right)}_{2\left(g\right)}\to {\mathrm{SrCrO}}_{4\left(s\right)}+{\mathrm{NiO}}_{\left(s\right)}+H_{2}O_{\left(g\right)}$$

This concept of using cheap material getters for vaporous chromium has shown potential in mitigating the effects of chromium poisoning for 40,000 to 50,000 h of system operation [52]. The science of getters may expand to the mitigation of other poisonous species like sulfur or silicon [51]. This may prove to be the best mitigation approach for SiO_2_ deposition, as other approaches are not well researched yet and only replacement of tectosilicate albite (NaAlSi_3_O_8_) sealant at the hydrogen electrode has been proposed [5,42].

### 3.2. Metallic Component Corrosion Suppression

Approaches similar to the growth of preferential oxide scales and deposition of coating varieties used to suppress cathodic poisoning have also been considered for dual atmosphere corrosion suppression. Preferential scale development of chromia and (Cr,Mn)_3_O_4_ spinel have positive effects in suppressing corrosion of metal interconnects [53]. When pre-oxidized, the ferritic stainless steels with substantial chromium (16–23%) form a dense, passivating chromia layer if the partial pressure of oxygen (*P*O_2_) of the oxidizing atmosphere is controlled. Forming the passivating Cr_2_O_3_ scale during a pre-oxidation has shown to be beneficial in mitigating expansive corrosion during dual atmosphere exposure. In air or high *P*O_2_ atmospheres, all constituents of the steel can oxidize, which often results in a chromia scale with cuboidal (Cr,Mn)_3_O_4_ crystallites and points of iron oxide growth [53]. Driving the *P*O_2_ to a lower limit will allow only chromia and (Cr,Mn)_3_O_4_ spinel to form. When exposed to dual atmosphere, the pre-oxidized samples out-performed the as-received in mitigating iron oxide overgrowth [54].

Using the Gibbs free energy for metal oxide formation (ΔG^0^), the *P*O_2_ in atmospheres can be calculated using the following relation:

(5) $$-\Delta G^{0}=-\mathrm{RTln}\left(PO_{2}\right)$$

In Equation (5), *R* is the universal gas constant in J/mol*K and *T* is temperature in *K*. This relation is the backbone of the Ellingham diagram of metal oxide formation, which is used to determine the equilibrium partial pressure of oxygen at a specific temperature and the ease of reduction of a metal oxide [54]. Oxygen activity can be approximated by its partial pressure using the following equation, as in Reference [54]:

(6) $$PO_{2}=\exp\left(\frac{1}{y}*\frac{2\Delta G^{0}}{\mathrm{RT}}\right)$$

In Equation (6), y is a coefficient respective of the reaction for a certain metal oxide M_x_O_y_, highlighted in Equation (7) [54]:

(7) $$\mathrm{xM}+\frac{y}{2}O_{2}\to M_{x}O_{y}$$

Equation (6) yields the range of oxygen partial pressure that will form a given oxide on its respective metal within the Ellingham diagram. Using the diagram, one can determine whether, at a certain temperature and *P*O_2_, a certain metal oxide will form or whether a metal will remain stable in the given conditions.

Chromia is considered a dense, passivating scale that hinders cationic mobility compared to the defect-heavy p-type FeO [54]. Cr and Fe are relatively similar in size, making their relative mobility through their respective oxides similar to mobilities in the opposing oxides. Sabioni et al. found that iron diffusion in chromia was hindered by thermodynamics, as chromia appeared to lower the oxygen potential at the metal/scale interface, which prevented iron in the metal from oxidizing [55]. According to Sabioni, the bulk cationic diffusion coefficient should vary with the oxygen pressure as (*P*O_2_)^3/16^ [55]. With *P*O_2_ considered, this would yield a diffusion coefficient in 1 atm oxygen equal to 5.6 times the diffusion coefficient in 10^−4^ atm oxygen [55]. It is clear from this that oxygen partial pressure works as a major driving force in oxide scale growth via influence on diffusion.

Talic et al. investigated the doped-spinel oxides of MnCo_2_O_4_, MnCo_1.7_Cu_0.3_O_4_, and MnCo_1.7_Fe_0.3_O_4_ deposited on interconnect steel Crofer 22 APU samples using electrophoretic deposition. The goal was to suppress corrosion in air while maintaining a higher conductivity than the commonly formed Cr_2_O_3_ and MnCr_2_O_4_ scales [56]. The spinel coatings reduced the parabolic rate of oxidation at the higher end of testing temperatures (800–900 °C); however, their mitigating effects diminished with a decrease in temperature. This was inconclusive on whether the coatings would ultimately improve corrosion resistance over cell operating time, as the area-specific resistance of the coated Crofer 22 APU was significantly lower than the uncoated alloy [56]. The concept of doped-spinel coatings is also used to combat Cr evaporation [57].

Coating varieties using reactive elements have been beneficial in providing corrosion resistance, much like the addition of alloying elements such as manganese and chromium within the alloy itself [58]. The reactive elements are rare earth in nature, usually zirconium, lanthanum, cerium, and yttrium [58]. These elements have a high oxygen affinity and a larger ion size than chromium, making them effective in promoting certain scale development. They have also been shown to improve chromia scale conductivity [59]. Their exact mechanism, known as the reactive element effect (REE), is unknown; however, Pint has discussed hypotheses in great detail based on early observations and hypotheses from Whittle and Stringer in 1980. These hypotheses include improved chemical bonding between oxide and alloy or a possible change in oxygen vacancy-assisted diffusion due to reactive element addition. However, these hypotheses are not yet conclusive. It was also noted by Pint that alloying elements may have the same effect and should also be considered in optimizing interconnect materials [60].

## 4. Conclusions

Solid oxide electrochemical systems serve as a promising means of providing clean and sustainable forms of energy in the near future. However, research in material development is needed to improve performance stability and increase system lifetime. The ongoing research efforts have proven effective in developing ways to mitigate the degradation of materials in these systems. Citing the nature of their operation, electrochemical systems are subjected to conditions which take a toll on the materials comprising them. Such degradations can be the result of harsh atmospheres, long operating times, elevated operating temperatures, and chemical compatibilities. These include poisoning of electrodes by gaseous species and corrosion of metallic cell interconnects caused by specific atmosphere exposure. This has led to various mitigation approaches that have examined materials used in systems, including material states, compositions, and coatings. Adsorbent gaseous species that threatened electrode operation were blocked by certain metal additions to the electrodes or captured by getters. Corrosion of metallic interconnects was mitigated through protective scale development and certain coatings. While materials challenges persist and degradation/corrosion continue to plague many electrochemical systems, research efforts and the overall potential of these systems has made them favorable in the quest for clean energy.
